# Homeotic transformations suggest mechanisms for rapid evolution diversification in *Drosophila* sex combs.

**DOI:** 10.17912/micropub.biology.000884

**Published:** 2023-08-14

**Authors:** Dawson Doucet, Nathan Friesen, Naomi Derksen, Megan Mulder, Stephen Ingram, Juan Nicolas Malagon

**Affiliations:** 1 Canadian Mennonite University, Winnipeg, Manitoba, Canada

## Abstract

Evolutionary innovations refer to the emergence of new traits, functions, or behaviors in organisms and lineages over time. Although research has demonstrated that such innovations can arise gradually or through small steps (Chouard 2010), the mechanisms by which rapid morphological diversification takes place remain poorly understood (Bailey *et al.* 2019). To explore this question, we used the evolution of sex combs, as a system (Ho *et al.* 2018). We used this male-specific row of leg bristles, comprising sex combs as a system, because it displays spectacular morphological diversification in a short time (Kopp 2011).

Homeotic mutations in the fruit fly, *Drosophila melanogaster,* are those which create modifications in one part of a fly to resemble another region. Here we describe effects of some of these mutations which transform the *D. melanogaster* fly sex comb morphology to closely resemble sex comb morphology in other species. These findings support previous research indicating that minor alterations to regulatory elements can play a significant role in explaining morphological evolution (Atallah *et al.* 2004). Thus, our results suggest that rapid diversification may not require starting from scratch, but rather may require minor modifications to the sex comb ground plan, which may account for its rapid morphological evolution (Lee *et al.* 2011).

**Figure 1. Leg homeotic transformations in Drosophila melanogaster mimic morphological variation observed during sex comb evolution:**
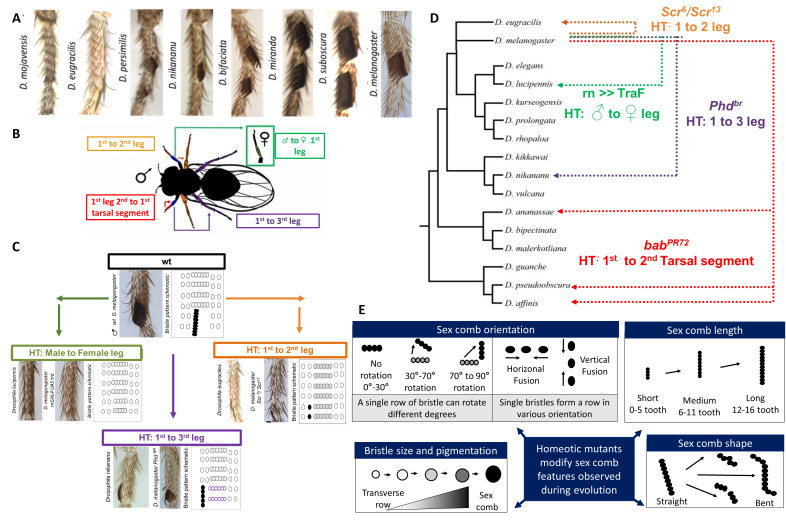
A) Morphological variation observed in sex combs among *Drosophila* species. Photographs of adult legs in various *Drosophila* species. Modified from Malagon et al. (2020). B) Image shows the four types of leg homeotic transformations examined in the study. C) Examples of sex comb chaetotaxy mimicry achieved through homeotic transformations of *D. melanogaster.* In the homeotic transformation from the 1 ^st^ to 2 ^nd^ tarsal segment (orange panel), although sex comb teeth were easily reproduced, transverse rows are missing (gray shaded ovals). In the homeotic transformation from the 1 ^st^ to 3 ^rd^ leg (purple panel), the transverse rows outlined in purple are not observed in *D. melanogaster,* and could potentially impede the rotation of the sex comb. D) Phylogenetic tree showing the relationship and homeotic transformations in *D. melanogaster* and *Drosophila* species. Dotted color lines showed similar sex comb phenotype. HT = homeotic transformation. Modified from Kopp (2011). E) Hypothetical model displaying how leg homeotic transformations can mimic sex comb evolution. Black circles represent sex comb teeth and empty circles represent the TR bristles.

## Description

Evolutionary innovations are new traits, functions, or behaviors that emerge in organisms and lineages over time (Kopp 2011) . Although some evolutionary innovations can occur due to gradual and slow morphological changes, some phenotypic traits can also diversify through small jumps and fast changes (Chouard 2010) . In addition, it is also extensively documented that some evolutionary innovations can appear, disappear, and re-appear again (Kopp 2011) . However, the mechanisms by which these evolutionary innovations can rapidly diversify remains poorly understood. Here, we asked how many genetic changes are necessary to mimic the phenotypic variation observed in a rapidly evolving trait, specifically in sex comb evolution.

A sex comb is a male-specific group of leg bristles located on the front legs of many *Drosophila* species (Kopp 2011; Ho *et al* . 2018). This row of bristles is used by males during courtship for various functions including to stimulate and hold onto the female during copulation (Kopp 2011) . *Drosophila* sex combs are excellent systems to study evolutionary innovations, displaying a spectacular range of morphological variation among species (Kopp 2011; Ho *et al.* 2018) ( Figure 1A ). In addition, as sex combs are secondary sexual traits, these leg bristles diversify relatively fast, displaying multiple examples where a sex comb trait can appear, disappear, and re-appear again in closely related species (Ho et al. 2018) . To study this rapid diversification, we previously suggested that these morphological changes can be explained by the presence of a basic “ground plan” in developing tarsal segments (Lee *et al* . 2011). This ground plan, represented by genetic and developmental processes, can be rapidly shifted during evolution by mutations producing homeotic transformations among various tarsal segments (Lee *et al* . 2011).

A homeotic transformation is a type of genetic mutation that causes the development of one body part or structure to be transformed into another body part or structure (Starling Emerald and Roy 1997). For example, the transformation of antenna into legs in fruit flies (Starling Emerald and Roy 1997; Struhl 1981). Almost a century ago, Goldschmidt used fruit fly homeotic transformations in the context of evolution, showing that a single mutation can produce a gradient of phenotypes by slightly modifying the basic ground plan of fly appendages (Dietrich 2003) . In previous work, we followed a similar approach, using homeotic transformations in leg tarsal segments, turning the 2 ^nd^ tarsal segment into the 1 ^st^ tarsal segment (Godt *et al.* 1993) ( Figure 1B ). We found that a single *Drosophila* *melanogaster* mutant, *bric à brac ^PR72^* ( *bab ^PR72^* ) is able to mimic the sex comb bristle patterns found in three other *Drosophila* species (Lee *et al* . 2011) ( Figure 1C -E). These findings raise the question of whether this mimicking potential is an exclusive feature of the mutant *bab ^PR72^* ? Or is it possible other homeotic mutations are also able to reproduce bristle patterns found in other *Drosophila* species?

To study the mimetic potential of homeotic transformations in sex comb evolution, we used genetic perturbations to change the normal identity of legs and tarsal segments. We selected homeotic transformations different from those achieved by *bab ^PR72^* (ta2 to ta1). The following are the three homeotic mutations studied: 1) T1 into T3 legs, 2) T1 into T2 legs, and 3) male into female legs ( Figure 1B -E). Then, adult legs from both *D. melanogaster* genetic perturbations were compared to other *Drosophila* species. The mimetic potential was evaluated based on parameters such as number of sex combs per tarsal segment, comb orientation, and comb pigmentation. The following *Drosophila* species were used for comparison: 1) *Drosophila* *nikananu* , 2) *Drosophila lucipennis* , 3) *Drosophila eugraciles* .

We found that homeotic mutants reproduce multiple sex comb features found in three different *Drosophila* species throughout the phylogeny ( Figure 1C -D). However, these genetic perturbations mimic different *Drosophila* sex combs from those previously reported using the mutant *bab ^PR72^* . For example, in the *phd ^br^* mutant, we found a sex comb accompanied by transverse row bristles from the top to the bottom of the first tarsal segment ( Figure 1C ).

In *D. melanogaster,* the sex comb typically originates from the most distal transverse rows on the first tarsal segment (ta1) (Kopp 2011) . As a result, transverse rows are not found in the most distal section of the ta1. However, in the *phd ^br^* mutant, both the sex comb and the most distal transverse row are present. This raises the question of the origin of the sex comb in this particular mutation. It is highly improbable that this ectopic sex comb is a result of rotation since the presence of distal transverse rows would obstruct any comb movement. In contrast, other *Drosophila* species exhibit a distinct trait in sex comb morphology, achieving vertical comb positioning without rotation ( Figure 1C ). These bristle patterns are observed in other *Drosophila* species such as *Drosophila* *nikananu* .

The findings reported here extend our previous work demonstrating the incredible potential of homeotic transformation to mimic sex comb evolution (Lee *et al* . 2011; Malagon 2013). This line of work suggests that changes in genetic regulatory factors play a key role in evolution (Atallah *et al.* 2004). However, this work is not suggesting that mutations in these genes are responsible for the rapid morphological diversification found among *Drosophila* sex combs, but the potential role of imperfect copies of morphological traits as an initial stage for evolutionary innovations.

## Methods

Flies were reared on yeast-cornmeal-molasses medium at 25°C. For image acquistion, adult legs were dissected, and mounted on cover slips 22X22 mm No 1 (VWR), and imaged in a light microscope (Olympus BX41M) with a Cool Snap camera U-CMAD (Photometrics) following the protocols described by Atallah (2008).

## Reagents

**Fly strains**

Four *D. melanogaster* genetic perturbations that produce homeotic transformation were studied, and their information is summarized in table 1. The UAS GAL4 system was also used to perturb sex comb development (Phelps and Brand 1998) . The driver chosen was rnGAL4-5 (BL. 7405), which is expressed in the distal region throughout 1, 2, 3, and 4 tarsal segments in the three legs. As a responder, UAS TraF was used (BL. 4590). Flies were reared on a yeast-cornmeal-molasses medium at 25°C.

The following *Drosophila* species were used for comparison: 1) *Drosophila* *nikananu* , 2) *Drosophila lucipennis* , 3) *Drosophila eugraciles* .

**Table:** 

***Drosophila melanogaster* Fly strains **	**Abbreviation**	**Source**
*Sex comb reduced* ^6^ / *Sex comb reduced* ^13^	* Scr ^6^ /Scr ^13^*	Anthony Percival- Smith
* Polyhomeotic distal ^br^*	* phd ^br^*	Bloomington 3952
Rotund GAL4	*rn-* GAL ^4-5^	Bloomington 7405
UAS Transformer	UAS TraF	Bloomington 4590
